# Commentary: The neural basis of human female mate copying: An empathy-based social learning process

**DOI:** 10.3389/fpsyg.2018.00397

**Published:** 2018-03-23

**Authors:** Severi Luoto, Meg J. Spriggs

**Affiliations:** ^1^English, Drama and Writing Studies, University of Auckland, Auckland, New Zealand; ^2^School of Psychology, University of Auckland, Auckland, New Zealand; ^3^Brain Research New Zealand, Auckland, New Zealand; ^4^Centre for Brain Research, University of Auckland, Auckland, New Zealand

**Keywords:** mate choice copying, social learning, facial processing, adaptation, byproduct, exapted learning mechanism

## Introduction

Our romantic partners are not only a source of intimacy for us and of genetic information for our offspring—their qualities also signal something about *us* to the external world. “Mate choice copying” is a form of social learning which allows an individual to learn from the information gathered and mate choices made by others (reviewed in Kavaliers et al., 2017; Anderson, 2018). Having examined the neural basis and functional specificity of the phenomenon, the authors of a recent experimental article suggested that mate choice copying may be “a domain-specific adaptation involving an empathy-based social-learning process that is also associated with reduced cognition” (Zhuang et al., 2017). We point out several shortcomings inherent in this conclusion, valuable though Zhuang et al.'s (2017) experimental data on the neurophysiological underpinnings of the trait may be.

## The neural substrates of mate choice copying

Zhuang et al. (2017) provide the first extensive exploration of the neural basis of mate choice copying in human subjects. The brain areas of significant activation identified by Zhuang et al. (2017) across task conditions are numerous and diverse, with the authors' conclusions based on significant activation of nodes within the empathy network. While the authors provide compelling explanations for the activations of additional networks in almost every experimental condition, the consistency with which some brain regions are activated across conditions is somewhat overlooked. For example, significant activation of the inferior frontal gyrus is recognized by the authors for opposing involvement in both the “higher cognitive functions” (including beauty judgements) associated with the “friend effect^1^,” and in the empathy and mirror neuron networks associated with mate choice copying (Zhuang et al., 2017). A simpler and more parsimonious explanation for the activation of this region across multiple contrasts could instead be based on its involvement in affective face processing (Green et al., 2015), which would be pertinent to both the friend effect and mate choice copying.

Paramount to the conclusions of Zhuang et al. (2017) is the activation of the Fusiform Gyrus/Fusiform Face Area (FFA), a region that is central to both face perception and expertise (Gauthier et al., 2000; Haxby et al., 2000). Perhaps unsurprisingly, this region was significantly activated across the majority of the examined statistical contrasts. Nevertheless, the authors place central emphasis on significant activation of this region in the pivotal three-way interaction between time, relationship context, and attractiveness, concluding that activation of the FFA here reflects the acquisition of “expertise” for the faces identified as potential mates (Zhuang et al., 2017). However, this is difficult to coalesce with both the acquisition time required for developing expertise (usually years; Gauthier et al., 2000), and the increased reaction times in this condition. Additional regions of significant activation in this three-way interaction include the hippocampus, thalamus, and superior parietal lobule—regions previously associated with recollection (Aggleton and Brown, 1999; Wagner et al., 2005). Recollection-based recognition is a relatively slow and effortful process involving the retrieval of contextual information (as opposed to familiarity-based recognition, which allows for quick decision making based on a “feeling of knowing”) (Aggleton and Brown, 1999). The recollection of contextual information (in this case, relationship status) is a central component of the authors' thesis, and as an alternative hypothesis, participants may be better able to recollect this contextual information as it is deemed more important when choosing potential mates. Because recollection is a relatively slow process, this would also account for the subjects' slower reaction time in the romantic partner context.

## Mate choice copying: a functionless byproduct or an exapted learning mechanism?

Bailey (2012) has argued that mate choice copying is a functionless byproduct of social learning, a view directly opposed to Zhuang et al.'s (2017) suggestion that mate choice copying is a domain-specific adaptation. Although some hallmarks of adaptation have been reported for mate choice copying, namely complexity, specificity, and possibly also reliability (Place et al., 2010; Vakirtzis, 2011; Zhuang et al., 2017), current evidence is insufficient to establish mate choice copying as an adaptation in humans (Street et al., 2018). The hallmarks of adaptation (Buss et al., 1998) that are yet to be confirmed for mate choice copying in humans are efficiency, capacity to solve adaptive problems, and evolvability (Vakirtzis, 2011; Witte et al., 2015).

If evidential support for these facets of mate choice copying as a hypothesized adaptation is eventually provided, it is a point of view of no small theoretical interest that mate choice copying be classified as an *exaptation*, not as an adaptation. An exaptation is a feature co-opted from existing mechanisms, enhancing fitness despite not being evolutionarily selected for that role (Gould and Vrba, 1982). Mate choice copying is one such example, since it has evolved from traits which were evolutionarily selected for other purposes, including neurophysiological and neurobiological substrates whose original functionality had little to do with mate choice copying (as reviewed by Kavaliers et al., 2017; Zhuang et al., 2017).

More accurately, mate choice copying could be classified as an *exapted learning mechanism*. A single learning mechanism may generate different cognitive mechanisms, each exhibiting specific design for performing a different task (Kruschke, 1992; Andrews et al., 2002). Under such circumstances, the learning mechanism has been *exapted* to a new problem and can be referred to as an *exapted learning mechanism* that shows design specificity (Andrews et al., 2002; see also Gangestad, 2010). Because an existing learning mechanism, social learning, has been functionally co-opted to solve another adaptive problem in mate choice copying, and because this new mechanism may show a degree of design specificity (Vakirtzis, 2011; Zhuang et al., 2017; yet see Street et al., 2018), its classification as an *exapted learning mechanism* is justified—but only to the extent that it fulfills the three outstanding criteria for adaptation: efficiency, capacity to solve adaptive problems, and evolvability. It is these facets that future research will have to empirically validate in order to establish whether mate choice copying in humans is an exapted learning mechanism or a mere functionless byproduct.

## Author contributions

SL and MS drafted the paper and reviewed it critically for intellectual content. SL and MS wrote and approved the manuscript.

### Conflict of interest statement

The authors declare that the research was conducted in the absence of any commercial or financial relationships that could be construed as a potential conflict of interest.

## Footnotes

^1^I.e., the finding that when a man was presented with a woman who was supposedly his friend (and not a romantic partner), he was rated as less attractive than when shown either by himself or with a romantic partner (Zhuang et al., 2017).
